# Malignant Priapism as a Result of Metastatic Thyroid Cancer: A Hard Reality

**DOI:** 10.1155/2021/5542092

**Published:** 2021-07-27

**Authors:** Ethan Vargo, Bryson Cook, Jason Lane, Eric Speakman, Neel Parekh

**Affiliations:** ^1^Cleveland Clinic Akron General Department of Urology, Akron General Avenue, Akron, OH 44307, USA; ^2^Northeast Ohio Medical University, 4209 St, OH-44, Rootstown, OH 44272, USA; ^3^Cleveland Clinic Akron General Department of Pathology, Akron General Avenue, Akron, OH 44307, USA

## Abstract

Metastasis to the penis is an extremely rare entity. Malignant priapism is defined as a persistent, nonsexual erection that is refractory to pharmacologic treatment, corporal aspiration, and surgical shunts. Furthermore, it is typically a hallmark of an advanced cancer that has metastasized, most commonly from regional organs like the prostate or bladder. We report an unusual case of malignant priapism in the setting of metastatic follicular thyroid carcinoma. To date, this is the second reported case of penile metastasis due to thyroid carcinoma and the first incidence of priapism secondary to follicular thyroid carcinoma metastasis.

## 1. Introduction

Primary cancer of the penis is a rare occurrence in North America and Europe, with rates approaching 1 in 100,000 men annually [1]. Metastases to the penis and malignant priapism, however, are exceedingly rare phenomena. Metastatic rectal adenocarcinoma to the penis was the first reported case of penile metastasis, as described by Eberth in 1870 [2]. While the vast majority of cases involving metastasis to the penis involve regional organs, there have been a limited number of cases originating from the head and neck region [3, 4]. Although a very rare occurrence, this report describes the phenomenon of metastasis to the penis resulting in a sustained erection and highlights the importance of considering metastatic disease as a differential diagnosis, especially in a patient with known malignancy presenting with priapism.

## 2. Case Presentation

The patient is a 73-year-old male with a past medical history of metastatic follicular thyroid carcinoma that was initially diagnosed in 2013. The patient underwent total thyroidectomy with radioactive iodine ablation in 2014. He was then found to have metastasis to the liver and underwent liver ablations in 2016 and 2018. In early 2020, the patient underwent positron emission tomography (PET) which demonstrated several metastatic sites involving the lung and bone and recurrent lesions within the liver. At this time, the patient underwent palliative radiation and was initiated on immunotherapy. Follow-up imaging in mid-2020 demonstrated regression of the previously identified pulmonary nodules.

The patient initially presented in August of 2020 to the urology service in the emergency department with a chief complaint of penile edema and pain for the previous few days. On physical examination, the penile shaft was significantly edematous, tender to palpation, and appeared to be erect. Based on the patient's history and physical exam, a diagnosis of priapism was made. A corporal blood gas was subsequently obtained. The corporal blood gas exhibited a pH of 6.999, consistent with ischemic priapism. Bedside corporal aspiration was then performed yielding a scant amount of thick, dark blood. Given the significant penile edema, it was difficult to ascertain the degree of improvement following corporal aspiration. The patient's pain, however, was well controlled, and he was discharged home with plans for close follow-up.

**Figure 1:**
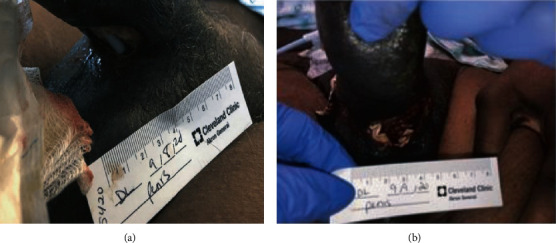
Physical exam findings prior to surgery demonstrated ventral urethral erosion and a visible Foley catheter (a). Postsurgical outcome after debridement and a visible Penrose drain at the base of ventral penile shaft (b).

The patient was admitted to the hospital two days later with shortness of breath, and he was found to have extensive metastatic disease to the lungs. Additionally, the patient also experienced new onset urinary retention and a Foley catheter was placed. Upon evaluation, the patient's physical exam was relatively unchanged from a few days prior. An inpatient pelvic MRI was ordered; however, it was unable to be obtained due to patient-reported claustrophobia. Within 24 hours of the Foley catheter placement, a new two-centimeter ulcerative lesion at the base of the ventral penile shaft was noted. There did appear to be erosion into the ventral bulbar urethra, as the Foley catheter that was previously placed for retention was now visible. Sloughing of the skin was present and the wound was foul smelling (Figure 1(a)). A decision was made to take the patient to the operating room urgently for exploration and debridement.

**Figure 2:**
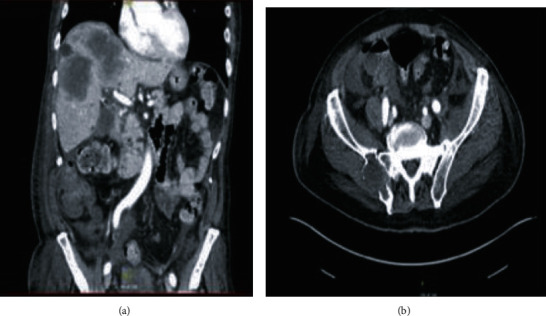
CT of the abdomen and pelvis with IV contrast demonstrating hepatic metastatic disease with an enlargement of the hepatic lobe mass. Right adrenal mass consistent with metastatic disease. Development of portacaval adenopathy consistent with metastatic disease (a). Expansile osteolytic right posterior ilium lesion (b).

**Figure 3:**
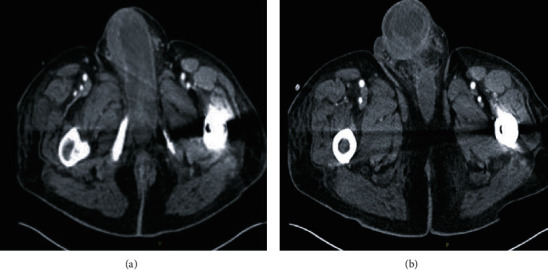
CT of the abdomen and pelvis with IV contrast obtained on postoperative day one demonstrating enlargement of the penis, likely consistent with metastatic, inflammatory, and postsurgical changes (a, b).

Initially, cystoscopy was performed to fully evaluate the urethra given the new ulcerative lesion on the ventral aspect of the penis eroding into the urethra. A flexible cystoscope was used as a rigid cystoscope was unable to bypass the area of necrosis. Once the gangrenous area was traversed and the bladder was entered, pan cystoscopy did not demonstrate any additional abnormalities in the urethra or bladder. A suprapubic catheter was placed. With the urine diverted, attention was then turned to the bulbar urethra. The wound was copiously irrigated and debrided. Bilateral penoscrotal decompression and corporal biopsies were performed. A Penrose drain was left, and the wound was packed with wet to dry gauze (Figure 1(b)). Minimal improvement of the patient's penile edema and priapism was noted on postoperative day one. A computed tomography (CT) scan of the abdomen and pelvis with IV contrast was obtained following surgery. This demonstrated several new hepatic lesions, a right adrenal mass, and portacaval adenopathy (Figure 2(a)). There was also enlargement of a right posterior ilium osteolytic lesion (Figure 2(b)). Additionally, enlargement of the penis at the base was noted (Figures 3(a) and 3(b)).

**Figure 4:**
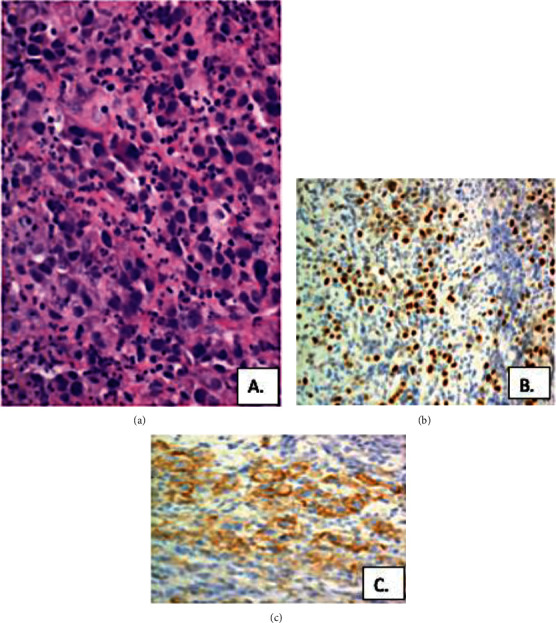
Histologic stains demonstrating hematoxylin and eosin stain of penile biopsies showing poorly differentiated carcinoma with dyscohesive malignant epithelioid cells demonstrating pleomorphism in a background of necrosis (a). Positive PAX-8 stain (b). Positive EMA stain (c).

Pathology from the intraoperative biopsies demonstrated poorly differentiated carcinoma with dyscohesive malignant epithelioid cells demonstrating pleomorphism in a background of necrosis (Figure 4(a)). Additionally, the cells stained positive for pan-keratin, paired box gene 8 (PAX-8) (Figure 4(b)), and epithelial membrane antigen (EMA) (Figure 4(c)) confirming a dedifferentiated or anaplastic process related to the patient's known history of metastatic follicular cell carcinoma of the thyroid.

Surgical options were discussed with the patient including penectomy; however, the patient was reluctant. Palliative radiation was deemed to be of minimal benefit by radiation oncology. The patient was ultimately discharged home, admitted to home hospice, and expired shortly after hospice initiation.

## 3. Discussion

Overall, metastasis to the penis is a rare phenomenon with fewer than 500 reported cases in the literature. Priapism, or a sustained erection for greater than four hours, represents 40% of the cases of metastatic disease to the penis [4]. Other manifestations of penile metastasis include penile masses, pain within the penis or perineum, voiding symptoms, and widespread swelling [5]. The most common sites of carcinoma origin in cases of metastasis to the penis include the bladder (28.6%), prostate (27.9%), and rectum-sigmoid (12.2%) [5]. Hypothesized pathophysiologic pathways leading to malignant priapism include direct tumor cell invasion of the corpora cavernosa, venous occlusion of the penis, and disruption of neural pathways [5].

Patients with penile metastasis do have slightly better survival when they undergo surgical intervention compared to those who undergo conservative measures like radiation and chemotherapy. Despite a greater than 80% mortality rate at one year for those presenting with penile metastasis, partial or total penectomy can be offered for improving short-term quality of life and minimizing local complications [4]. Our patient had new evidence of widely metastatic disease on presentation as depicted via imaging in Figure 2 and likely would not have benefited from further surgical management given his very poor performance status and limited lifespan.

Immunohistochemical staining in the setting of a rare disease process can provide valuable information in the determination of disease origin. In our patient's case, intraoperative biopsy of the penis was consistent with poorly differentiated carcinoma with dyscohesive malignant epithelioid cells, which stained positive for pan-keratin, EMA, and PAX-8. The transcription factor, PAX-8, orchestrates embryonic development within Mullerian organs, the kidney, and the thyroid [6]. In one study, PAX-8 was detected in 100% of patients with primary follicular thyroid cancer, and the authors concluded that PAX-8 is both sensitive and specific for cell staining in cases of metastatic tumors of thyroid origin [6]. Another study demonstrated significance in the expression of EMA in patients with follicular thyroid carcinomas [7]. The PAX-8- and EMA-positive staining of the poorly differentiated cells in our patient's penile biopsies suggests that the patient's malignant priapism was a result of his metastatic follicular thyroid cancer. Furthermore, the presence of metastatic thyroid tumor cells within our patient's penile corpora highlights the hematogenous and potentially distant pattern of metastasis that follicular thyroid carcinoma commonly demonstrates [8].

Only one other case of thyroid cancer metastasizing to the penis has been reported in the literature, highlighting the rarity that is associated with metastasis from a distant organ. That patient had a history of metastatic follicular thyroid cancer that had metastasized to the bone and pelvis. Additionally, the patient developed a nodule on the proximal penile shaft and subsequently underwent total penectomy with the creation of a perineal urethrostomy and ultimately expired secondary to his metastatic disease [4]. With the very limited number of case reports describing metastasis to the penis and only one other case report describing metastasis from follicular thyroid carcinoma, it is difficult to derive an algorithm for clinical and surgical management of these patients. One clinical takeaway is understanding the widespread and terminal nature of a malignancy by the time penile metastasis is identified. In those who present with priapism without a history of malignancy, the clinician should consider metastatic priapism due to an undiagnosed regional or distant organ metastasis.

## 4. Conclusion

Metastasis to the penis and malignant priapism are very rare phenomena; however, they should be considered especially in a patient with a known history of malignancy. Limited clinical and surgical options exist given the known disseminated nature of disease on presentation.
